# Effect of Glass Fiber Incorporation on Flexural Properties of Experimental Composites

**DOI:** 10.1155/2014/542678

**Published:** 2014-07-17

**Authors:** Rodrigo Borges Fonseca, Aline Silva Marques, Karina de Oliveira Bernades, Hugo Lemes Carlo, Lucas Zago Naves

**Affiliations:** ^1^Dental School, Federal University of Goiás, Praça Universitária, s/n, Setor Universitário, 74605-220 Goiânia, GO, Brazil; ^2^Department of Restorative Dentistry, Health Sciences Center, Federal University of Paraíba, 58051-900 João Pessoa, PB, Brazil; ^3^Piracicaba Dental School, State University of Campinas, 13414-903 Piracicaba, SP, Brazil

## Abstract

This study evaluated the effect of fiber addiction in flexural properties of 30 wt% silica filled BisGMA resin (FR) or unfilled Bis-GMA (UR). Ten groups were created (*N* = 10) varying the resin (FR or UR) and quantity of glass fibers (wt%: 0, 10, 15, 20, and 30). Samples (10 × 2 × 1 mm) were submitted to flexural strength test following SEM examination. Data were analyzed by two-way ANOVA, Tukey, and Student *t*-test (*α* = 0.05). Results for flexural strength (MPa) were FR-groups: 0% (442.7 ± 140.6)^C^, 10% (772.8 ± 446.3)^ABC^, 15% (854.7 ± 297.3)^AB^, 20% (863.4 ± 418.0)^A^, 30% (459.5 ± 140.5)^BC^; UR-groups: 0% (187.7 ± 120.3)^B^, 10% (795.4 ± 688.1)^B^, 15% (1999.9 ± 1258.6)^A^, 20% (1911.5 ± 596.8)^A^, and 30% (2090.6 ± 656.7)^A^, and for flexural modulus (GPa) FR-groups: 0% (2065.63 ± 882.15)^B^, 10% (4479.06 ± 3019.82)^AB^, 15% (5694.89 ± 2790.3)^A^, 20% (6042.11 ± 3392.13)^A^, and 30% (2495.67 ± 1345.86)^B^; UR-groups: 0% (1090.08 ± 708.81)^C^, 10% (7032.13 ± 7864.53)^BC^, 15% (19331.57 ± 16759.12)^AB^, 20% (15726.03 ± 8035.09)^AB^, and 30% (29364.37 ± 13928.96)^A^. Fiber addiction in BisGMA resin increases flexural properties, and the interaction between resin and fibers seems better in the absence of inorganic fillers increasing flexural properties.

## 1. Introduction

For over than 30 years fibers have been used as structural reinforcement for dental resins [1], including carbon, polyethylene, aramid, and glass fibers. Carbon fibers, despite its high strength, have a great aesthetic drawback due to its natural black color [2]. Glass fibers display high tensile strength and aesthetic appearance and have been widely studied in terms of strengthening effect [3–6] and interaction with composite or acrylic resins [5, 7, 8] in order to manufacture root posts [9, 10].

The effectiveness of fiber reinforcement depends on many factors, including the resin material, the quantity, length, shape, orientation, and adhesion properties of fibers [11]. Recent studies suggest that most failures occur at the interaction of fibers with surrounding resins [2], which can be theoretically solved by fiber silanization before resin impregnation [12, 13], and additional heat application in silanated fibers [14].

Studies have shown the relationship between the amount of fibers in polymer matrix and the flexural strength of tested reinforced materials [15–17]. According to the law of mixtures the flexural strength increases linearly as more fibers are included in resins [5]. Research shows that greater quantity of glass fibers also result in higher flexural modulus [16]. The use of 22.5 wt% 3 mm short glass fiber in 22.5 wt% photopolymerizable methacrylate resins (Bis-GMA, TEGDMA, and polymethylmethacrylate) and 55 wt% inorganic fillers resulted in high fracture strength for severely damaged postrestored incisors [18].

Since the inclusion of inorganic fillers increases resin viscosity limiting the addiction of fibers into resin, the hypothesis driven in the present research is that a pure BisGMA-based resin (without inorganic filler particles) would enable the inclusion of greater quantity of glass fibers resulting in higher flexural properties. The aim of this study was to evaluate the effect of short 3 mm long glass fiber addiction in flexural strength and flexural modulus of photopolymerizable 30%(wt) silica filled BisGMA composite resin (FR) or unfilled Bis-GMA resin (UR).

## 2. Materials and Methods

A factorial design was employed to create experimental groups (*N* = 10) according to the interaction of factors in study: the quantity of 3 mm glass fibers added (wt%), in five levels, and the type of resinous material, in two levels, as seen in Table 1. The unfilled resin (UR) was composed of a photopolymerizable BisGMA: bisphenol-A-glycidyl dimethacrylate (Angelus, PR, Brazil) and the filled resin (FR) had 75 wt% of BaAlSiO_2_-radio-opacity-filler in BisGMA (Natural Flow, Nova DFL, RJ, Brazil). Resins were used as provided by manufacturer and then the groups were created by manual incorporation of 3 mm short glass fibers into resins.

### 2.1. Flexural Strength Tests

Bar-shaped specimens (10 mm × 2 mm × 1 mm) were made in a half-split stainless steel mold between transparent Mylar sheets [19]. For the controls, no fiber was mixed to the resins and, for the other groups, 10, 15, 20, and 30 wt% of 3 mm long glass fibers were used. Glass fibers were soaked in a silane solution for 1 min (Silano, Angelus, PR, Brazil) and mixed to resin materials, being inserted into the mold and light polymerized by 40 s with a LED device at 850 mw/cm^2^ (Radi-e, SDI, Australia). After that, specimens were finished manually on 600, 1000, and 1200-grit SiC sandpapers with running water, for 10 seconds on each side [14]. Samples were stored in distilled water at 37°C for 24 hr until flexural tests begin [5].

A universal testing machine (Instron 5965, Instron Co., Canton, USA) was employed for a three-point bending test, accomplished with 8 mm test span, at 0.5 mm/min, with a central load application until fracture takes place [19]. The maximum load at fracture was recorded in Newton and load-deflection curves were recorded with PC software (Bluehill 2, Instron Co.). Flexural strength (FS) and flexural modulus (FM) were calculated with the following formula: FS = 3*P* · *L*/2*w* · *h* ^2^, FM = *S* · *L* ^3^/4*w* · *h* ^3^, where

 
*P*: maximum load at fracture,
 
*L*: test span (8 mm),
 
*w*: specimen width (2,0 mm),
 
*h*: specimen thickness (1 mm),
 
*S*: the stiffness (N/m). S: P/d,
 
*d*: deflection corresponding to P applied load at the straight-line portion of the load-deflection curve.

Fractured specimens were silica dehydrated and sputter-coated with gold (MED 010; Balzers Union, Balzers, Liechtenstein) for observation in the Scanning Electron Microscope (DSM 940A; Zeiss, Oberkoshen, Germany).

Data were submitted to a factorial analysis with a general linear model procedure to analyze the interaction between factors in study. After that statistical analysis was completed with Kolmogorov-Smirnov test of normal distribution and 1-way analysis of variance (ANOVA) followed by Tukey HSD test or Student *t*-test (pairwise comparisons). For all tests, groups were considered statistically different at *α* = 0.05.

## 3. Results

Tables 2 and 3 show FS and FM results, respectively. The factorial analysis showed interaction between factors (*P* = 0.0001). Further analysis showed that within FR groups the 20%wt glass fiber addiction resulted in the highest flexural strength, which was similar to 10-FR and 15-FR. The 0-FR (control group) showed the lowest results, followed by 30-FR and both were similar to 10-FR group, but just the 30-FR was similar to 15-FR. UR groups showed an increase on flexural strength according to the percentage of glass fiber addiction. The 0-UF group showed the lowest results, followed by 10-UR; the 30-UR showed the highest flexural strength, which was similar to 15-UR and 20-UR.

Paired comparisons between FR and UR groups with similar wt% of glass fibers showed that, only at 10%wt addiction of glass fibers, UR and FR groups were similar to each other. The UR without fiber reinforcement (control group) had lower FS than FR; however all the other fiber reinforced UR groups achieved statistically higher flexural strength than FR groups (*P* < 0.05).

For flexural modulus within FR groups, the 20-FR presented the highest value, which was similar to 10-FR and 15-FR. The 0-FR showed the lowest results, followed by 30-FR and both were similar to the 10-FR group. For UR groups the highest FM was presented by 30-UR, which was similar to 15-UR and 20-UR. The control 0-UR presented the lowest FM, similar to 10-UR. The 10-UR was considered statistically similar to 15-UR and 20-UR.

Paired comparisons between FR and UR groups with similar wt% of glass fibers showed the same behavior on flexural strength: 10%wt glass fiber UR and FR groups were similar to each other and all the other groups were considered statistically different (*P* < 0.05).

**Figure 1:**
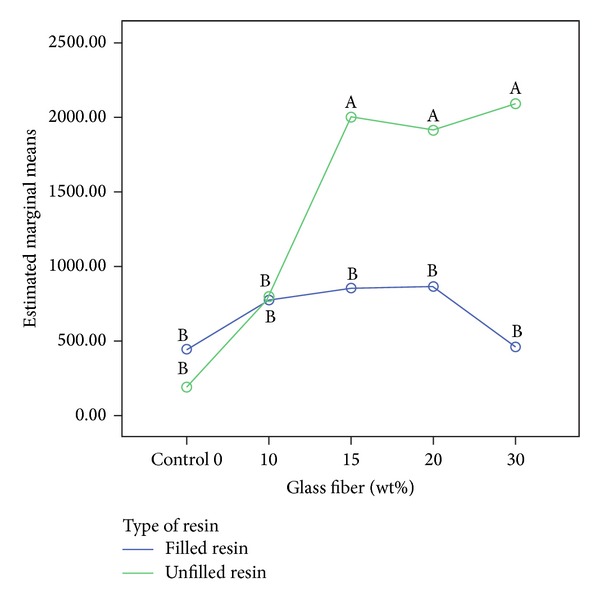
Flexural strength (MPa) for all groups compared together. Green line refers to unfilled groups and blue line to filled groups. Different letters mean statistical significant differences with *P* < 0.05.

**Figure 2:**
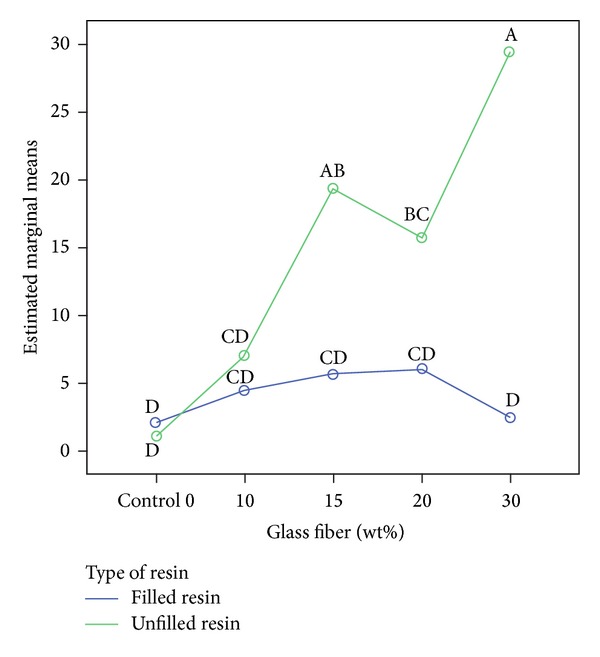
Flexural modulus (GPa) for all groups compared together. Green line refers to unfilled groups and blue line to filled groups. Different letters mean statistical significant differences with *P* < 0.05.

One-way ANOVA and Tukey HSD test for the comparison of all groups together showed the tendency for unfilled resin groups with high percentages of glass fibers displaying higher flexural strength and flexural modulus (Figures 1 and 2).

**Figure 3:**
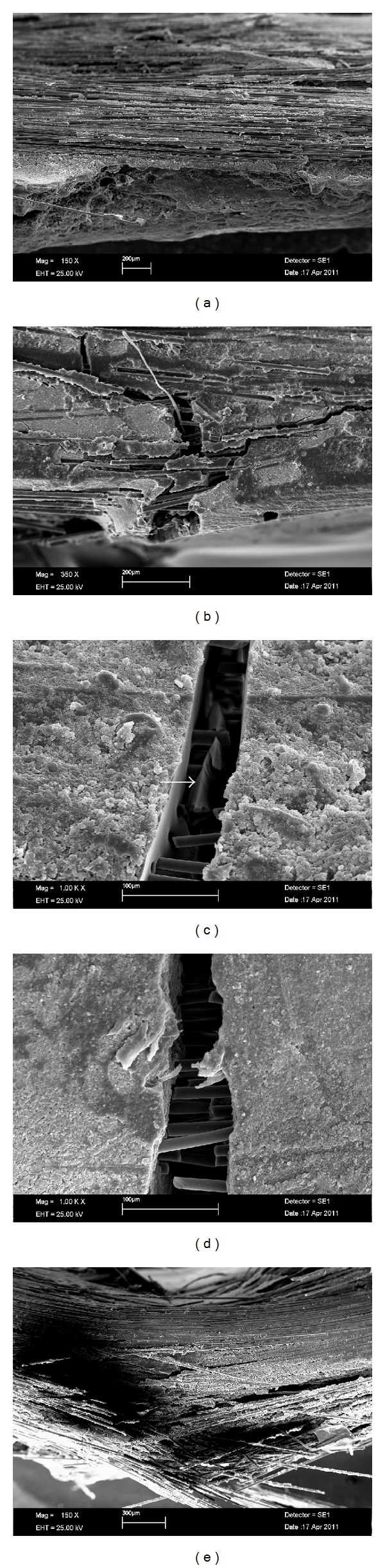
SEM images of groups. (a) SEM (original magnification: 150x) of UR-20 showing clear interaction between fibers and resin and homogeneous distribution of these components in the composite; (b) SEM of 20-FR (original magnification: 150x) showing interaction fibers and resin and heterogeneous distribution of these components in the composite; (c) SEM of 10-UR (original magnification: 1000x) showing the crack development region with a clear cohesive fracture around glass fibers within the crack (arrow); (d) SEM of 10-FR (original magnification: 1000x) showing the crack development region without resin cohesive fracture around glass fibers within the crack; (e) fiber dislodgement on 30-FR (original magnification: 150x) as a result of few resin matrix to keep fibers together.

SEM analysis (Figure 3) showed better fiber wetting by resin on UR groups (Figure 3(a)), resulting in greater homogeneity, as seen by fiber and resin distribution in the composite; the more homogeneous composite seemed to enable an “easier” interaction between resin and fibers and consequently better protection against fracture development (Figure 3(a)). In spite of the fact that FR groups showed resin impregnation over glass fibers, a heterogeneous distribution of fibers and resin was clearly seen (Figure 3(b)). Experimental groups with lower %wt of glass fibers showed cohesive fractures of the resinous component on UR groups (Figure 3(c)), but not on FR groups (Figure 3(d)).  Figure 3(e) shows the dislodgement of fibers as a consequence of interaction failure due to high %wt of fibers within FR.

## 4. Discussion

Glass fiber posts possess mechanical properties similar to dentin, which enables more favorable stress distribution into remaining tooth structure, preventing root fracture [20]. However, the most reported problems with fiber posts are related to coronal post fractures [21] or debonding [20], which requires the development of stronger posts and better adhesion procedures. The first requirement can be achieved with the development of fiber reinforced composite resins, as shown by Garoushi et al., 2009 [18].

Composite resin reinforcement with glass fibers tends to produce better mechanical flexural properties than filler reinforcement [17], but, as stated, there should be an optimal relationship between resin and fibers enabling adhesion between resin matrix and the fiber reinforcement material. Poor adhesion between glass fibers and methacrylate resins leads to an intrinsic requirement for the use of longer fibers, in order to increase mechanical friction. The present study showed BisGMA resins are able to wet glass fibers no matter the percentages of fibers included in unfilled or inorganic particle filled resins. However, it could be seen that a homogeneous distribution of these components within the composite is desirable since the filled resin reduced flexural strength and modulus when greater quantities of fibers were added to the resin. Thus this study partially agrees with the hypothesized expectations, since it was possible to include similar quantity of fibers in either unfilled or filled resins but the increase in mechanical flexural properties was more pronounced on the unfilled groups.

In order to evaluate the strengthening effect of fibers within resin materials, 3-point bending flexural strength tests have been employed. According to Della Bona et al. 2004 [22], these tests are supported by ISO standards and mechanically create stresses within materials enabling the evaluation of strengthening mechanisms. Internal specimen defects are in part derived from heterogeneous polymerization. The ISO4049/2000 [23] recommends 25 mm (±2,0) × 2 mm (±0,1) × 2 mm (±0,1) specimen dimensions; however, the dental photopolymerization devices' tip usually measures 10 mm in diameter, being necessary more than one photopolymerization cycle to cover all the specimen length. Consequently, a heterogeneous polymerized material will be created. According to Pick et al. [19], smaller specimens (10 mm × 2 mm × 1 mm) must be used to eliminate this problem, as in the present study.

By means of a fiber fragmentation test it has been estimated that the critical E-glass fiber length varies from 0.5 to 1.6 mm [24]. This critical length enables maximum stress transfer from the resin matrix to fiber reinforcement [24] and also makes each fiber to behave as individual crack stoppers [25]. Clinically, the use of smaller fiber length has shown increased resin wear and reduced fracture resistance [26]. Garoushi et al. 2006 [16] observed 2 to 5 mm long fibers resulting in similar values for flexural strength and modulus and compressive resistance, although the greatest values obtained in their study were in samples with 5 mm long fibers. However, 3 mm long fibers exceed the fiber critical value and enable a multidirectional reinforcement, being the reason for which studies have used this fiber length [18, 25, 27, 28].

In the present study, the use of 3 mm long fibers increased flexural strength, especially in unfilled resin groups. Figure 3(c) shows a cohesive resin fracture inside the crack, with glass fibers around the fracture, which seems to be evidence that stress transfer occurred but the resin itself was not able to resist. Figure 3(d) shows the same crack development but without cohesive resin fracture possibly due to the use of filled resin. Both figures are from 10%wt reinforced groups, which showed the first increase in flexural properties after fiber reinforcement (Figures 1 and 2). At higher %wt fiber reinforcement, unfilled groups increased flexural properties significantly more than the filled resin groups.

Studies show that the quantity of fibers is directly related to the flexural mechanical properties of fiber reinforced resins (FRC) [16, 29] and filler addiction does not improve these properties [27]. With a FRC composed by 22.5%wt fiber in 22.5 wt% resin matrix and 55 wt% silica filler, Garoushi et al. 2008 [28] showed acceptable depth of cure and microhardness for clinical use. They have also shown higher load bearing capacity, when this material was used to manufacture posts, than those with fiber post and composite core [18] and when used to manufacture premolar crowns [27]. In the present study, reduced FS and FM for 30-FR group could then be explained by a negative effect of more %fibers on polymerization of this filled resin, but more research is necessary to support this assumption.

Poor fiber impregnation may be a result of using a more viscous resin, leaving empty spaces that accumulate oxygen, which inhibits resin polymerization. This fact produces a flexural weak FRC [24]. In the present study, during specimen manufacturing it could be noted that FRC polymerization was visually more difficult in FR-groups, especially in groups with high %wt fibers. In 30-FR group, there was few resin matrix to keep fibers together, causing fiber dislodgement (Figure 3(e)) and reducing significantly the flexural properties.

When comparing the results of this work with data from literature it is possible to conclude that fracture strength was significantly improved. By using 22%wt of 3 mm long glass fibers in BisGMA-TEGDMA (60% bisphenol-A-glycidyl dimethacrylate-40% triethylenglycoldimethacrylate), Garoushi et al. 2006 [16] showed 146 MPa and 8.1 GPa for FS and FM, respectively. With similar composition but 20%wt glass fibers, the group 20-UR achieved 1911.55 MPa and 15.72 GPa for the same variables. Possibly, the reason for such a difference was the application of a silane coupling agent on glass fibers and also the different employed specimen dimensions. Figures 3(a) and 3(b) show interaction between resin and fibers but only at Figure 3(a) a homogeneous spatial fiber distribution can be seen, and this was accounted for the observed higher FS and FM of most of unfilled versus filled groups.

The optimum glass fiber/resin/filler proportion was not investigated on this research and needs further work. As seen, high values for flexural strength and flexural modulus can be obtained if inorganic fillers are not included in the composite but several other properties need to be investigated for establishing good mechanical properties and handling characteristics.

## 5. Conclusions

The inclusion of short glass fibers (3 mm long) in a BisGMA resinous matrix increases the flexural strength and flexural modulus of the material, and the absence of inorganic filler promotes better interaction/homogeneity with the fibers, leading to greater increase in the analyzed parameters. Inclusion of 30 wt% of fibers into filled resin reduced mechanical properties but the same into unfilled resin had the opposite behavior.

## Figures and Tables

**Table 1:** Experimental groups and codes.

Quantity of glass fibers (%)	Type of resinous materials	
	Filled resin (FR)	Unfilled resin (UR)
0	0-FR	0-UR
10	10-FR	10-UR
15	15-FR	15-UR
20	20-FR	20-UR
30	30-FR	30-UR

**Table 2:** Flexural strength mean and standard deviation (MPa) for the interaction between %wt of glass fibers and type of resinous material. Statistical comparisons by ANOVA following Tukey-HSD test and paired comparisons by Student *t*-test (*α* = 0.05).

Quantity of glass fibers (%wt)	Type of resinous materials	
	Filled resin	Unfilled resin
0	442.78 ± 140.65^Ca^	187.76 ± 120.37^Bb^
10	772.81 ± 446.33^ABCa^	795.48 ± 688.19^Ba^
15	854.78 ± 297.35^ABb^	1999.91 ± 1258.65^Aa^
20	863.46 ± 418.05^Ab^	1911.55 ± 596.88^Aa^
30	459.55 ± 140.59^BCb^	2090.61 ± 656.79^Aa^

Different capital letters (vertical analysis) and lower case letters (horizontal analysis) mean statistical significant differences with *P* < 0.05.

**Table 3:** Flexural modulus mean and standard deviation (GPa) for the interaction between %wt of glass fibers and type of resinous material. Statistical comparisons by ANOVA following Tukey HSD test and paired comparisons by Student *t*-test (*α* = 0.05).

Quantity of glass fibers (%wt)	Type of resinous materials	
	Filled resin	Unfilled resin
0	2.06 ± 0.88^Ba^	1.01 ± 0.71^Cb^
10	4.48 ± 3.02^ABa^	7.03 ± 7.86^BCa^
15	5.69 ± 2.79^Ab^	19.33 ± 16.75^ABa^
20	6.04 ± 3.39^Ab^	15.72 ± 8.03^ABa^
30	2.49 ± 1.34^Bb^	29.36 ± 13.92^Aa^

Different capital letters (vertical analysis) and lower case letters (horizontal analysis) mean statistical significant differences with *P* < 0.05.
